# An Efficient Disinfectant, Composite Material {SLS@[Zn_3_(CitH)_2_]} as Ingredient for Development of Sterilized and Non Infectious Contact Lens

**DOI:** 10.3390/antibiotics8040213

**Published:** 2019-11-07

**Authors:** V.A. Karetsi, C.N. Banti, N. Kourkoumelis, C. Papachristodoulou, C.D. Stalikas, C.P. Raptopoulou, V. Psycharis, P. Zoumpoulakis, T. Mavromoustakos, I. Sainis, S.K. Hadjikakou

**Affiliations:** 1 Inorganic Chemistry laboratory, Department of Chemistry, University of Ioannina, 45110 Ioannina, Greece; basilikikaretsi92@gmail.com; 2 Medical Physics Laboratory, Medical School, University of Ioannina, 45110 Ioannina, Greece; nkourkou@uoi.gr; 3 Department of Physics, University of Ioannina, 45110 Ioannina, Greece; xpapaxri@uoi.gr; 4 Laboratory of Analytical Chemistry, Department of Chemistry, University of Ioannina, 45110 Ioannina, Greece; cstalika@uoi.gr; 5 Institute of Nanoscience and Nanotechnology, NCSR “Demokritos”, 15341 Athens, Greecev.psycharis@inn.demokritos.gr (V.P.); 6 Institute of Biology, Medicinal Chemistry and Biotechnology, National Hellenic Research Foundation, 11635 Athens, Greece; pzoump@eie.gr; 7 Organic Chemistry Laboratory, Department of Chemistry, National and Kapodistrian University of Athens, 15571 Athens, Greece; tmavrom@chem.uoa.gr; 8 Cancer Biobank Center, University of Ioannina, 45110 Ioannina, Greece; isainis@cc.uoi.gr

**Keywords:** biological inorganic chemistry, antimicrobial material, zinc(II) complexes, sodium lauryl sulphate, citric acid, hydrogel

## Abstract

The [Zn_3_(CitH)_2_] (**1**) (CitH_4_= citric acid), was dispersed in sodium lauryl sulphate (SLS) to form the micelle of SLS@[Zn_3_(CitH)_2_] (**2**). This material **2** was incorporated in hydrogel made by hydroxyethyl-methacrylate (HEMA), an ingredient of contact lenses, toward the formation of pHEMA@(SLS@[Zn_3_(CitH)_2_]) (**3**). Samples of **1** and **2** were characterized by UV-Vis, ^1^H-NMR, FT-IR, FT-Raman, single crystal X-ray crystallography, X-ray fluorescence analysis, atomic absorption and TG/DTA/DSC. The antibacterial activity of **1**–**3** as well as of SLS against Gram-positive *(Staphylococcus epidermidis (St. epidermidis)* and *Staphylococcus aureus (St. aureus))* and Gram-negative *(Pseudomonas aeruginosa (PAO1)*, and *Escherichia coli (E. coli))* bacteria was evaluated by the means of minimum inhibitory concentration (MIC), minimum bactericidal concentration (MBC) and inhibitory zone (IZ). **2** showed 10 to 20-fold higher activity than **1** against the bacteria tested. Moreover the **3** decreases the abundance of Gram-positive microbes up to 30% (*St. aureus*) and up to 20% (PAO1) the Gram-negative ones. The noteworthy antimicrobial activity of the obtained composite **3** suggests an effective antimicrobial additive for infection-free contact lenses.

## 1. Introduction

Microbial keratitis often leads to blindness, unless if it is immediately treated. Ocular infection is either caused by misuse contact lens or a wide variety of pathogens [1,2,3]. However, contact lenses have become a major etiology for microbial keratitis, nowadays [4,5,6,7]. Therefore, the development of contact lenses built by long term or permanent, non-infected materials is a research, technological and financial issue challenge. The soft contact lenses are generally made by the hydrogel, poly (2-hydroxyethyl methacrylate) (pHEMA) and they have already been investigated for ocular delivery of several drugs [2,8,9].

Gram-positive bacteria are the major contributor of bacterial ocular infections, with the most commonly reported belong to the genus Staphylococci such as *Staphylococcus epidermidis* (*St. epidermidis*) and *Staphylococcus aureus* (*St. aureus*) [3]. Among Gram-negatives, on the other hand, frequent isolate from ocular infections include *Pseudomonas aeruginosa* (*PAO1*) and *Escherichia coli* (*E. coli*) [3].

Zinc is an essential element for the microorganisms since it is involved in vital cellular reactions even at its low endogenous concentrations. The antimicrobial properties of Zn^2+^ and its complexes have been known since long time now [10,11,12,13,14].

SLS is an anionic surfactant and detergent with microbicidal activity, due to its ability to induce protein denaturation [15]. It is use in the personal care products industry (e.g., toothpastes and shampoos) [15,16]. It has been classified as readily biodegradable, with no any concern in respect to human health by United Nations Environment Programme (UNEP) [17]. SLS forms micelles by encapsulating bioactive materials adjusting their hydrophilicity [18]. This makes possible the loading non water soluble antimicrobial agents to be incorporated in hydrogels, acting, by thus, as a vehicle [18].

In the course of our studies on the development of new antimicrobial materials for medical devices [11,19,20,21], the slightly water soluble [Zn_3_(CitH)_2_] (**1**) which contains CitH_4_ (C_6_H_8_O_7_) was encapsulated in a micelle of SLS with the aim to enhance its dispersion inside a hydrogel. The compound and its composite SLS@[Zn_3_(CitH)_2_] (**2**) were characterized by their physical properties and spectral data. Either in solid state or in solution the concentration of 1 in the composite material is evaluated. This is used for the determination of MIC, MBC, IZ, biofilm elimination concentration (BEC) and the % cells viability upon treated with **3**, which is impossible otherwise to be determined. The antimicrobial activities of **2** and its components SLS and **1** were evaluated against the Gram-positive or negative microbes. The influence of **2** against the formation of biofilm of *St. aureus* was also evaluated. The composite **2** was incorporated in hydroxyethyl methacrylate (HEMA), an ingredient of contact lenses to form pHEMA@(SLS@[Zn_3_(CitH)_2_]) (**3**) which was evaluated for its antibacterial efficiency. Although zinc citrate in combination with SLS has been already studied for the inhibition of plague [22], here we described the extensive characterization of the composite material derived by the combination of [Zn_3_(CitH)_2_] (**1**) with SLS for the first time. Moreover, this work mainly aims to the development of sterilized and non infectious contact lens, where the composite SLS@[Zn_3_(CitH)_2_] (**2**)is an ingredient by its dispersion into pHEMA.

## 2. Results

### 2.1. General Aspects

**Scheme 1:**
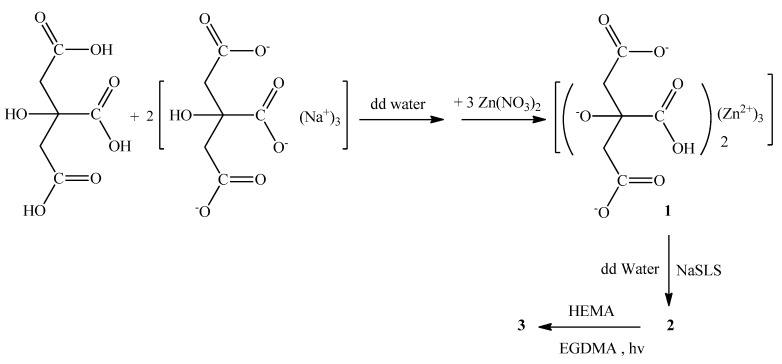
Reaction scheme for the preparations of compounds **1**–**3**.

Compound **1** was synthesized as follows: trisodium citrate reacted with CitH_4_ in 1:2 molar ratio, in 2 mL dd-water (Scheme 1). Disodium citrate solution (2 mmol) was added to a clear solution of Zn(NO_3_)_2_ (3 mmol) in dd water (Scheme 1) under stirring for 10 min.

Crystals of **1** were grown by slow evaporation of the solution. The formula of **1** was initially determined by spectroscopic methods and its molecular structure was confirmed by single crystal X-ray diffraction analysis. The structure of **1** was confirmed as previously reported by Che et al. [23] (**1**: a = 6.144(4) Å, b = 14.577(4) Å, c = 9.585(4) Å, β = 102.50(2)°, V = 838(1) Å^3^; the reported: monoclinic, space group P2_1_/c, a = 6.1552(13) Å, b = 14.546(3) Å, c = 9.581(4) Å, β = 102.66(2)°, V = 836.9(4) Å^3^ [23]). Compound, **1** was prepared previously, under hydrothermal conditions which include heating at 150 °C for 20 h [23]. However, this is synthesized here, at ambient condition upon few minutes stirring of its components. The variation in preparation conditions and the absence of detailed spectroscopic studies of **1** led us to collect these data for its structure evaluation. The crystals of **1** are air stable when they are stored in darkness at room temperature. The **1** is poorly soluble in H_2_O, and it is insoluble in organic solvents.

### 2.2. Characterization of **1**

#### 2.2.1. Solid State Studies

**Figure 1:**
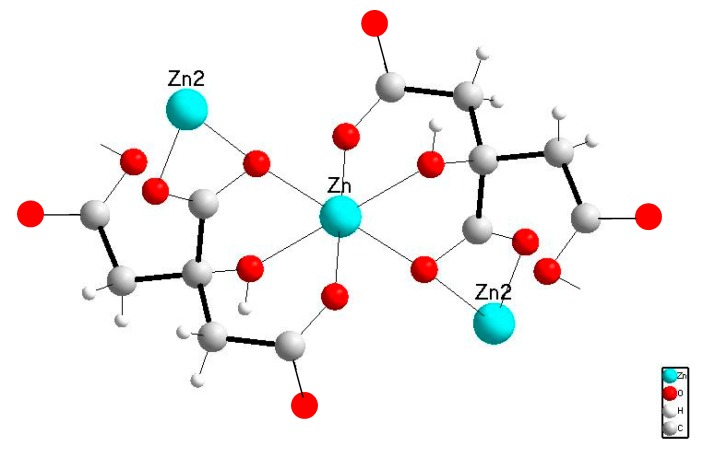
Molecular diagram of **1**.

*Crystal and molecular structure of***1**: The crystal and molecular structure of **1** is known [23], however the refinement of its X-ray diffraction data was proceeded for the verification of the product. The formula of the building block of the entire compound **1** is shown in Figure 1.

Two different environments are identified around the zinc atoms in this structural unit. One is bonded to the citrate ligand via hydroxyl group, the central carboxyl group and one of the terminal carboxyl groups of the ligand in a distorted octahedral arrangement. In the second one, the zinc ion is coordinated to a water oxygen atom as well as to six carboxyl oxygen atoms of three carboxyl groups forming seven-coordinated metal center.

*Vibrational spectroscopy*: The FT-IR spectrum of **1** shows vibration bands at 1400 cm^−1^ and at 1557 cm^−1^, which are assigned to symmetric (*v*_s_) and asymmetric (*v*_as_) bands of (COO^−^) respectively (Figure S1). The corresponding vibration bands of the trisodium citrate (CitHNa_3_) are observed at 1394 cm^−1^ and at 1560 cm^−1^, respectively. The difference in Δ*v*[*v*_as_(COO-)-*v*_s_(COO-)] values of CitHNa_3_ (166 cm^−1^) and **1** (157 cm^−1^) suggests the coordination of the ligand in to the metal [24].

#### 2.2.2. Solution Studies

**Figure 2:**
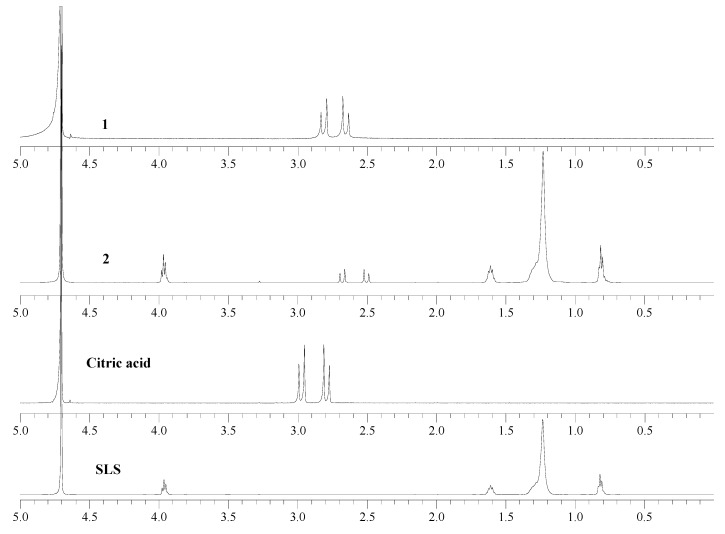
Spectra of **1**–**2** and SLS and CitH_4_ in D_2_O.

*Stability studies*: The stability of **1** in water solution was tested by UV-Vis and by ^1^H-NMR spectroscopies. UV-Vis spectra were recorded initially and for a period of 24 h. No differences were observed between the initial UV-Vis spectrum and the corresponding ones after 24 h suggesting the stability of **1** in solution for 24 h, at least (Figure S2).Moreover the double signals of methylene protons (H^b^ and H^b’^) of CitH_4_ are assigned at 2.99–2.95 and 2.81–2.77 ppm (Figure 2) and they are shifted in the case of **1** at 2.83–2.79 and 2.67–2.63 ppm, respectively, indicating the coordination of the metal to the ligand. The assignment of the ^1^H-NMR spectrum of **1** is based on its crystal structure data suggesting the retention of the structure in solution.

On the other hand, despite of the already examples on the effect of pH on the stability and biological activities of Zn(II) complexes [13,14], no such study was performed in case of **1**, since our work aims in the formation of a water soluble micelle of **1** which will be used as an ingredient in the hydrogel **3**.

### 2.3. Preparation and Characterization of Micelle

*Determination of critical micelle concentration (CMC)*: Due to the low solubility of the **1** in aqueous media, the micelle **2** is formed, using the SLS as surfactant. In order to assert the formation of the **2**, the determination of the CMC was performed via potentiometric titration at 36 °C [25] and the graph of conductivity values vs [SLS]/[**1**] is drawn (Figure S3). Above the CMC value, surfactant molecules self-agglomerate to form the micelles [26]. Upon the formation of the micelles, a turning point (change of slope) is observed in the graph [26]. The CMC in case of **2**, was determined as follows: a solution of SLS (2 × 10^−2^ M) in ddH_2_O was added to 20 mL ddH_2_O solution of **1** (8.9 × 10^−4^ M) in portions of 1 mL, while the conductivity values were being recorded subsequently. The CMC value is obtained at [SLS]:[**1**]molar ratio of 8.7 (Figure S3). Thus, by mixing its components in a molar ratio [SLS]:[**1**] = 8.7 the composite **2**, is formed. This ratio corresponds to a concentration of 5.7 mM of SLS. The corresponding concentration for the formation of micelle of free SLS is 8.3 mM. This is expected since upon the formation of micelles of drug with surfactants thereby decrease the CMC of surfactant [27].

#### 2.3.1. Solid State Studies

##### Vibrational Spectroscopy

FT-IR: The IR spectra of the **1**, SLS and **2** were recorded (Figure S4). The vibration band at 1557 cm^−1^ in the spectrum of **1** is attributed to ν_asym_ of the COO− of the CitH_4_ and it is absent from the corresponding spectrum of SLS. The presence of this band in the spectrum of **2** suggests the encapsulation of **1** in SLS.

**Figure 3:**
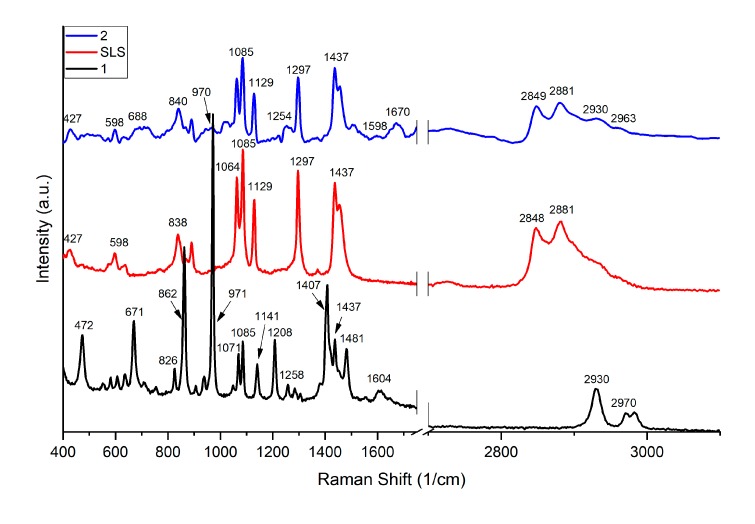
Raman spectrum of the micelle of **2** compared to corresponding ones for sodium lauryl sulphate (SLS) and 1. The spectra are artificially offset for clarity. The data for **2** was baseline corrected due to fluorescence.

*FT-Raman*: Raman spectra of **1**, **2**, and SLS are shown in Figure 3. The spectrum of SLS show intense Raman bands related to the ν(C–C) stretching vibration in the region between 1000 and 1150 cm^−1^, a strong peak at 1297 cm^−1^ related to ν(SO_4_) stretching and at 1437 cm^−1^ due to δ(CH_2_) deformations. The strong bands at 2848 and 2881 cm^−1^ are attributed to the α-CH_2_ scissoring and (CH_2_)_n_ symmetric stretch.

In the spectrum of compound **1**, the antisymmetric stretching vibration of the carboxylate groups v_as_(COO−) yields the band at 1604 cm^−1^. The corresponding symmetric stretching v_s_(COO−) generates the intense Raman line at 1407 cm^−1^. These peaks are typically used as guidelines to determine the coordination mode of the ligand. Together with the absence of a band at ~1735 cm^−1^ assigned to the free carboxylic acid group, the asymmetric and symmetric COO− stretching are characteristic for the ligand coordination scheme. The strong Raman band at 971 cm^−1^ is assigned to ν(C-C) mode. At 671 cm^−1^, the δ(COO−) deformational mode is shown. Metal–ligand (Zn–O) vibrations appear as an intense Raman band at 472 cm^−1^ while vibrations related to ν(CH_2_) and ν(C–H) modes are visible as strong Raman lines at 2930, 2970, and 2983 cm^−1^. The majority of the remaining bands cannot be assigned unambiguously. However, the presence of the COO−, and the C–H stretching bands in the spectrum of **2** shifted to 688, and 1598 cm^−1^, and 2930 cm^−1^ respectively, confirms the encapsulation of 1 in SLS.

##### Thermo Gravimetric Analysis of the Composite 2

*Differential scanning calorimetry (DSC)*: Encapsulation of **1** in SLS aims in the adjustment of its solubility in polar or non-polar solvents and through this, in the control of its releasing in the cytoplasm or other intracellular organelles.

**Figure 4:**
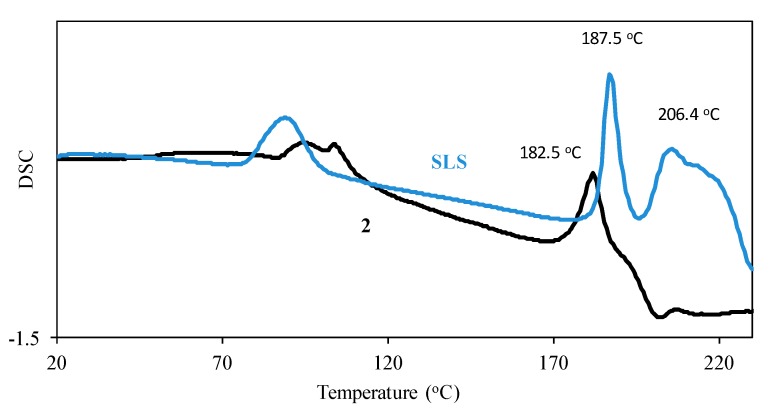
DSC thermograms of SLS and **2**.

To confirm whether **1** interacts with SLS in the solid state to give a mixture, DSC studies were carried out. DSC thermodiagrams of SLS and **2** are shown in Figure 4. The broad endothermic peak between 70–120 °C may be attributed to the boiling of the water in the SLC or in compounds **1** and **2**. Two sharp endothermic transitions at 187.5 and 206.4 °C are observed in the DSC diagram of SLS, while only one at 182.5 °C for **2**. The melting point of SLS is 204–207 °C, while the corresponding one of **1** is above 240 °C. The absence of any transition at 205–207 °C in the DSC diagram of the composite **2** suggests the formation of a composite instead of a mixture.

*Thermal Decomposition*: TG/DTA analysis was performed under nitrogen. The composite **2** decomposes with four exothermic steps at 36.2, 92.5 °C due to the elimination of the solvents and at 198.5 and 521.0 °C (Figure S5A) with total mass loss of 64.06%. The SLS decomposes in two exothermic steps that occur at 213.4 and 424.2 °C (Figure S5B) with total mass loss of 70.80%.

**Figure 5:**
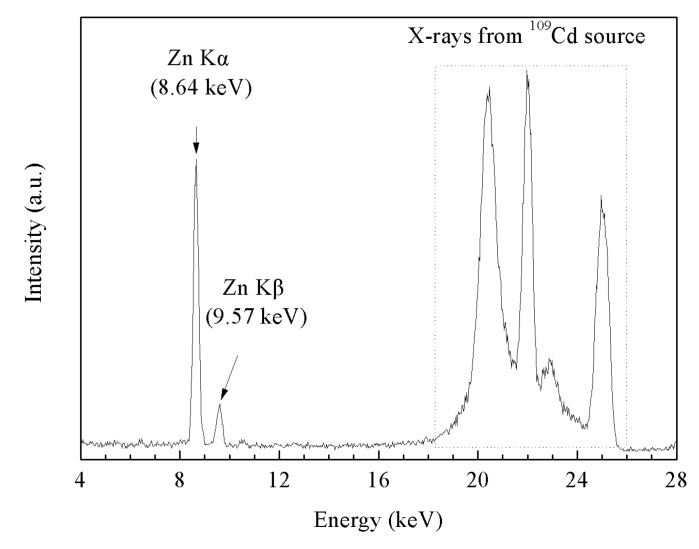
A typical XRF spectrum acquired from sample **2**, showing the characteristic Zn Kα and Zn Kβ X-ray peaks.

*X-ray fluorescence spectroscopy*: The XRF spectrum of **2** confirms the presence of Zn in the **2** which is indicative of the encapsulated **1** into the micelle (Figure 5).

The content of micelle **2** in zinc was determined by XRF spectroscopy and was 6.4± 0.8 % wt, which corresponds to dispersion of **1** in the SLS by 18.9% wt. This leads to a [SLS]:[**1**] molar ratio equal to 8.6 which is in agreement to the value obtained for CMC (8.7).

#### 2.3.2. Solution Studies

*Atomic Absorption*: In order to ascertain the dispersion of the **1** in its composite **2**, graphite furnace atomic absorption spectroscopy (GFAAS) was employed. The mass content of the zinc in the micelle **2** was determined to be 25.06% *w*/*w* which leads to a molar ratio [SLS]:[**1**]= 6.0. The differences in the molar ratios resulted for the contents of the composite, depending to the method used (CMC: 8.7, X-ray fluorescence spectroscopy: 8.6, atomic absorption: 6.0) should be attributed to the physical state in which the measurements were carried out (solid state for CMC and X-ray fluorescence spectroscopy; solution state for the Atomic absorption) and to the accuracy of them.

*^1^H NMR studies*: The ^1^H NMR spectra of **2** and SLS are shown in Figure 2. The multiple resonance signals at 0.83–0.80 ppm are assigned to the protons of the (-CH_3_)^ω^ group of the SLS [28], while the multiple signals at 1.28–1.23 ppm and 1.62–1.58 ppm are attributed to its (-CH_2_-)_9_ and (CH_2_)^β^ protons. The multiple signals at 3.98–3.94 ppm are assigned to the protons of the (CH_2_)^α^ group of the SLS. It is noteworthy to mention that the two double signals at 2.69–2.66 and 2.52–2.49 ppm which are assigned to the methylene protons of the CitH_4_ [28], underline a strong upfield shift towards the corresponding signals observed in the free ligand (2.81–2.77 ppm) and in complex **1**(at 2.83–2.79 and 2.67–2.63 ppm). These shifts are indicative of the coordination of the **1** to SLS confirming the formation of the composite instead of a mixture in case of **2** as evidenced by DSC studies.

It is noteworthy to mansion, here, that species distribution for Zn^2+^—citrate within various pH values involve the identification of the [Zn(cit)]^−^, [Zn(cit)H], [Zn(cit)_2_]^4−^ and [Zn_2_(cit),H_−2_]^4−^ in water, which is suggesting pH dependence [29]. However, DSC studies demonstrated the formation of the composite material **2** only, and therefore any shifts in the NMR spectra, in single pH value, can be assigned to the interaction between **1** and SLS in the composite material 2.

*Drug loading in pHEMA*: pHEMA cross-linked with ethylene glycol dimethacrylate (EGDMA), is the basis of many types of daily wear soft contact lenses [9]. In order to evaluate the sterilizing efficiency of contact lenses which contain antimicrobial agents, **2** was dispersed in pHEMA to form compound **3**. The content of the hydrogel in **1** is 2 mM (given that its content in the micelle was found 25.1%). Discs, of 10 mm diameter were subsequently cut, cleaned from monomers and stored either in sterilized NaCl 0.9% *w*/*w* solution or they dried at 50 °C.

### 2.4. Biological Studies

*Antibacterial Effect of **1***-***2***, *SLS andCitH_4_ on the growth of microbial strains*: The antimicrobial potency of **1**–**2**, SLS, and CitH_4_, against Gram-positive (*St. epidermidis* and *St. aureus*) and Gram-negative (*PAO1* and *E. coli*) is evaluated by the mean of minimum inhibitory concentration (MIC) [30]. MIC is defined as the lowest concentration needed for the inhibition of the bacterial growth [30]. The MICs values of **1**, **2**, and SLS against microbes studied here are summarized in Table 1 (Figures S6–S9). The CitH_4_ exhibits no activity against either Gram-positive or negative bacteria (Figures S6–S9). Thus, the composite **2** exhibits up to 21-fold higher antibacterial potency than free **1** and 5-fold stronger than free SLS, in the case of *St. aureus*. However, in the case of Gram-negative bacteria the composite exhibits either similar (*PAO1*) of 2-fold higher (*E. coli*) activity than free SLS, suggesting its sensitivity towards Gram-positive bacteria. Moreover, the encapsulation of the non-active **1**, into the SLS, creates a composite material with significant strong activity against Gram-positive bacteria (*St. epidermidis* and *St. aureus)* (Table 1).

*Minimum bactericidal concentration testing*: The lowest concentration of an antibacterial agent that can eliminate the 99.9% of the bacterial inoculum is known as minimum bactericidal concentration (MBC) [31]. The MBC values were: 201.7 μΜ(**1**), 13.4 μΜ (**2**) and 100 μΜ (SLS) against *St. epidermidis* (Figure S10), Table 1), while the corresponding MBC values against *St. aureus* are: >250 μΜ (**1**) 12 μΜ (**2**) and 60 μΜ (SLS) respectively (Figure S11) (Table 1). The MBC values of CitH_4_ are higher than 250 μΜ against *St. epidermidis* and *St. aureus*. The MBC values of **1–2**, SLS, and CitH_4_ are higher than 250 μΜ against *PAO1* and *E. coli* (Figures S12 and S13) (Table 1).

Moreover, the MBC/MIC ratios of **1**–**2** and SLS against *St. epidermidis* and the corresponding ones of **2** and SLS against *St aureus* are lower to 2 (Table 1). When MBC/MIC value is less than 2, the compound is classified into bactericidal one, indicating that it kills 99.9% of the microorganisms, while if MBC/MIC ≥ 4 then it is bacteriostatic and it inhibits but not kill the organism [32]. Therefore the agents **1**–**2** and SLS are classified in bactericidal ones against Gram-positive bacteria.

**Figure 6:**
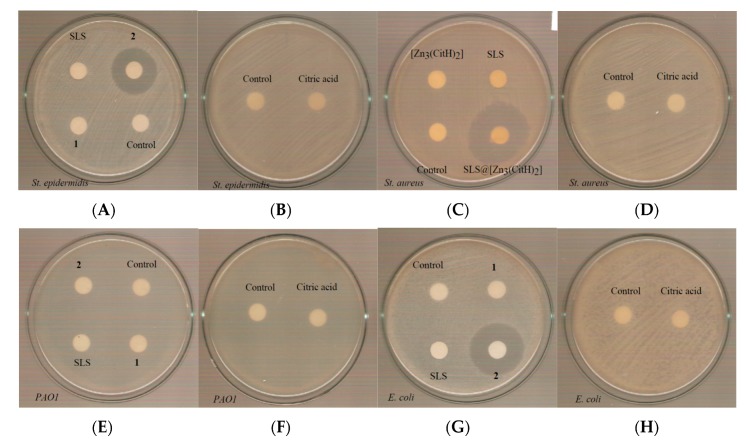
Inhibition zones which were grown in *St. epidermidis* (**A**,**B**), *St. aureus* (**C**,**D**), *PAO1* (**E**,**F**) and *E. coli* (**G**,**H**) for **1**, **2**, SLS, and CitH_4_.

*Determination of the inhibition zone (IZ) through agar disk-diffusion method*: The agar disk-diffusion method was used in order to survey the sensitivity of the microorganism to the antibacterial agent studied here [33]. The diameter inhibition zones of bacterial growth caused by of **1**-**2**, SLS, and CitH_4_ at concentration of 1 mM against *St. epidermidis*, *St. aureus*, *PAO1*, and *E. coli* were measured after 20 h (Table 1, Figure 6). The corresponding ones caused by **2** are 23.0 mm, 30.5 mm, and 26.5 mm against *St. epidermidis*, *St. aureus*, and *E. coli* respectively. No inhibition zone was observed upon treatment of *PAO1* with the composite material. Moreover, no inhibition zone was formed for of **1**, SLS, and CitH_4_ against all tested bacteria. Therefore, the presence of SLS enhances the antibacterial properties of **1** upon the formation of the composite, in agreement with the MIC experiment. The microbe strains may classified in three categories according to the size of IZ, caused by an antimicrobial agent in their agar dilution culture: (i) strains where the agent causes IZ ≥17 mm are susceptible, (ii) those where the agent creates IZ between 13 to 16 mm (13 ≤ IZ ≤ 16 mm) are intermediate, while (iii) those where the agent causes IZ ≤ 12 mm, the microbes are considered as resistant strains [34]. Therefore, the strains of *St. epidermidis*, *St. aureus,* and *E. coli* respond susceptible against **2** [34].

*Effects on biofilm formation by **2***: The adhesion of bacterial cells to surfaces is the initial stage of the formation of biofilm. Thus, bacterial colonies are attached in a surface and they are protected in a polysaccharides matrix. It is estimated that 80% of all clinical infections are biofilm related. Especially, the ophthalmic biofilm infections are difficult to treat. The necessity of the surgical removal of the infected tissue which is then following in the untreated cases contains high risk for corneal blindness. The biofilm elimination can be achieved by applying metallodrugs or surfactant molecules [35].

The effect of **2** against biofilm formation of *St. aureus*, was studied by the biofilm elimination concentration (ΒΕC) using crystal violet assay [9]. The ΒΕC is defined as the concentration required to achieve at least a 99.9% reduction in the viability of biofilm bacteria. The composite was able to inhibit the biofilm formation, reducing the 100% of biomass at 700 μΜ, against *St. aureus* (Figure S14). The BEC value of ciprofloxacin, a known antibiotic for the bacterial keratitis is 897 μΜ for the *St. aureus* [9]. Thus, the composite is 1.3 times more efficient against biofilm than the commercially available antibiotic ciprofloxaxin.

**Figure 7:**
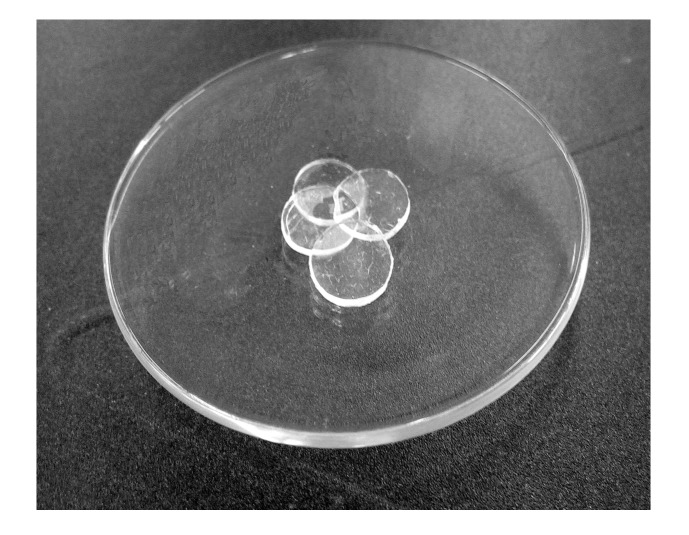
Dried discs of **3**.

*Antimicrobial activity of****3***: Since the composite exhibits antimicrobial activity against the tested strains, this prompts us to load it into pHEMA for the development of new non-infectious contact lens (Figure 7). The **3** and pHEMA discs were placed in tests tubes which contain 5 × 10^5^ cfu/mL of *St. epidermidis*, *St. aureus*, *PAO1*, and *E. coli* microbes.

**Figure 8:**
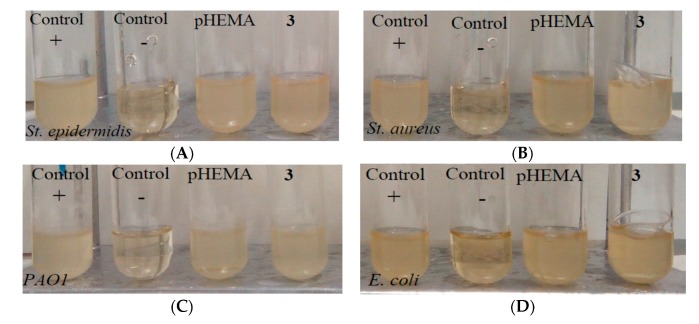
Bacteria viability *St. epidermidis* (**A**), *St. aureus* (**B**), *PAO1* (**C**) and *E. coli* (**D**) upon their incubations above discs of pHEMA or **3.**

The calculated bacterial % viability of *St. epidermidis*, *St. aureus*, *PAO1*, and *E. coli* upon their incubation with **3** discs for 20 h is 72.5±22.2%, 71.0±18.0%, 81.2±6.5%, and 93.5±7.7% respectively (Figure 8). On the contrary, no influence in the bacterial viability was observed against these bacterial strains, upon their treatment by discs of pure pHEMA (Figure 8). Moreover, the **3** is more active against positive than negative bacterial strains. This is in agreement with the MIC values found for the **2**.

**Figure 9:**
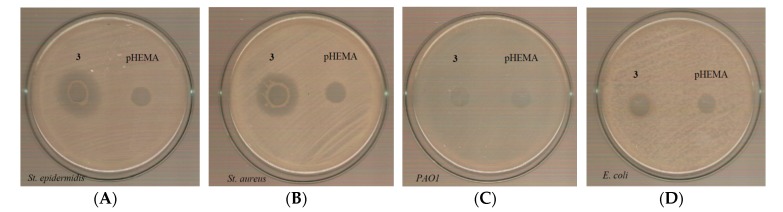
Inhibition zones of pHEMA and **3** against *St epidermidis* (**A**), *St. aureus* (**B**), *PAO1* (**C**), and *E. coli* (**D**).

Discs of pHEMA or **3** with diameter 10 mm, were placed to petri agar dishes and the IZs were determined. The inhibition zones of **3** against *St. epidermidis* and *St. aureus*, were 14.0 mm and 17.0 mm respectively, while no inhibition zones were developed against *PAO1* and *E. coli* (Table 1, Figure 9). Moreover, no inhibition zones were developed when pHEMA was used against all tested strains (Figure 9). Given that the IZs, which were developed when paper discs instead of pHEMA ones, were loaded by 2, were significantly greater, a low releasing of 2 from pHEMA, can be concluded.

## 3. Conclusions

This work aims in the development of sterilized and noninfectious contact lens. The encapsulation of **1** in SLS towards **2** was attempted in order to increase its solubility in polar solvents particularly into water. The composite material **2** is proven to be an efficient disinfectant. Thus, a stronger activity towards Gram-positive than negative bacteria is exhibited by the composite **2**, which is up to 21-fold higher than free **1** and 5-fold stronger than free SLS, in the case of *St. aureus*. The **2**, behaves as bactericidal material, while the Gram-positive bacteria as susceptible against it. Also, the **2**, is 1.3 times more efficient against biofilm than the commercially available antibiotic ciprofloxacin, which is used in ocular infections. Material **2** is loaded into pHEMA for the development of the new non-infectious contact lens ingredient **3**. The calculated % bacterial elimination of *St. epidermidis*, *St. aureus*, *PAO1* and *E. coli* bacteria, when they are incubating with **3** discs, is 27.5%, 29.0%, 18.8% and 6.5% respectively. In conclusion **3**is an effective candidate towards the development of new non-infectious contact lens.

## 4. Materials and Methods

*Materials and instruments*: All solvents used were of reagent grade. Tryptone and soy peptone were purchased from Biolife. Agar was purchased from Sigma-Aldrich. Sodium cloride, D(+)-glucose, di potassium hydrogen phosphate trihydrate were purchased from Merck. Citric acid, trisodium citrate, sodium lauryl sulfate, and zinc nitrate (Sigma-Aldrich, Merck, Darmstadt, Germany) were used without further purification. Dimethylsulfoxide (DMSO) and boric acid were from Riedel-de Haen. Melting points were measured in open tubes with a Stuart Scientific apparatus and are uncorrected. FT-IR spectra in the region of 4000–370 cm^−1^ were obtained from KBr discs, with a Perkin-Elmer Spectrum GX FT-IR spectrophotometer. Raman spectra were obtained with 785 nm solid state excitation laser coupled to a focusing fiber optic probe with output power of approximately 90 mW, 5 s accumulation time, in the 400–3150 cm^−1^ spectral range. Stokes photons were detected with a 2048 pixel thermoelectrically cooled CCD. The ^1^H-NMR spectra were recorded on a Bruker AC 400 MHz FT-NMR instrument in D_2_O solution. A UV-1600 PC series spectrophotometer of VWR was used to obtain electronic absorption spectra. XRF measurement was carried out using an Am-241 radio isotopic source (exciting radiation 59.5 keV). Conductivity measurements were performed at 36 °C in H_2_O solutions with a WTF LF-91 conductivity meter.

*Synthesis and crystallization of **1***: A clear solution of 0.5 mmol CitH_4_ mono-hydrate (0.105 g) and 1 mmol of trisodium citrate di-hydrate (0.294 g) were stirred in 2 mL of distilled water. Next, 3 mmol Zn(NO_3_)_2_ 6 H_2_O (0.892 g) were added in the solution and were stirred. White crystals of **1** suitable for X-ray analysis were grown from slow evaporation of the solution after 2 days.

**1:** White crystals, MW= 574.258, yield= 0.240 g; melting point: >240 °C. Elemental analysis found:C, 25.35; H, 1.72%; Calc. for C_12_H_10_O_14_Zn_3_, 25.02; H, 1.75%; IR (cm^−1^), (KBr) of **1**: 1559vs (ν_asym_(-COO-)), 1541s, 1399vs (ν_sym_(-COO-)), 1258s (ν(C-OH)), 1139w, 1083s(ν(C-OH)), 1070s (ν(C-OH)), 934s (ν_stretching_(C-C)), 903s (ν_stretching_(C-C)), 861s (ν_deformation_(COO)); ^1^H NMR(ppm) in D_2_O:2.67–2.63 and 2.83–2.79 (H^b^, H^b’^)

*X-ray Structure Determination*: Data collection was performed with a Rigaku R-AXIS SPIDER Image Plate diffractometer, using graphite-monochromated Mo-Ka(*λ*= 0.71073 Å) radiation at 160 K. Data collection (ω-scans) and processing (cell refinement, data reduction and Empirical absorption correction) were performed using the CrystalClear program package [36].

*Micellar Synthesis of **2***: 0.051 g (0.09 mmol) of **1** and 0.164 g (0.56 mmol) of **SLS** were dissolved in 100 mL of distilled H_2_O and stirred to clearness at 36 °C. The clear solution was then allowed for solvent evaporation within few days. The micelles were collected as white aggregates. IR (cm^−1^), (KBr) of **2**: 1560w, 1457w, 1246w, 1215vs, 1061s, 901w, 828s, 721w, 630s, 590s; IR (cm^−1^), (KBr) of **SLS**: 1250w, 1216vs, 1060s, 970w, 827s, 712w, 630s, 590s. ^1^H NMR (ppm) in D_2_O **2**:0.81, 0.83 (_ω_CH_3_), 1.22, 1.24, 1.26 (CH_2_)_9_, 1.59, 1.61, 164 (_β_CH_2_), 2.53–2.49, 2.70–2.66 (H^b^, H^b’^), 3.96, 3.97, 3.99 (_α_CH_2_).

*Synthesis of **3***: Hydrogel of pHEMA with incorporated **2** is obtained as follows: 2.7 mL of HEMA were mixed with 2 mL of double distilled water (ddw), which contains **2** (2 mM) and 10 µL of EGDMA. The solution was then degassed by bubbling with nitrogen for 15 min. TPO initiator (6 mg) was added to the solution and mixed for 5 min at 800 rpm. The solution was poured into the mold and was then placing under a UV mercury lamp (λmax=280 nm), 15 watt, where photopolymerization was occurred, for 40 min. Un-reacted monomers were removed, by immersing the gel in boiling water for 15 min. Discs with 10 mm diameter were cut by a puncher, and they were washed by immersion in dd water and NaCl 0.9%, HCl 0.1 M. For the antimicrobial activity tests, the hydrogel discs were stored in sterilized NaCl 0.9% *w*/*w*.

*Atomic Absorption*: A solution of 0.00134 g **2** was dissolved in 500 μL ultra pure nitric acid. The detection of zinc mass in the sample was performed with a graphite furnace atomic absorption spectrophotometer (GFAAS).

### Biological Studies

Bacterial Strains: For the antibacterial experiments the strain of *Staphylococcus epidermidis* (ATCC^®^ 14990™), *St. aureus subsp. aureus* (ATCC^®^ 25923™), *P. aeruginosa PAO1* and *Escherichia coli Dh5a* (*E. coli*) were used.

*Antibacterial effects of **1***–***2****and SLS on the growth of microbial strains*: This study was performed according standard procedure which is also described elsewhere [9,11,20,21]. Briefly, the bacterial strains were streaked in trypticase soy agar. The plates were incubated for 18–24 h at 37 °C. Three to five isolated colonies are selected of the same morphological appearance from the fresh agar plate using a sterile loop and transfer into a tube containing 2 mL of sterile saline solution. The optical density at 620 nm is adjusted to 0.1 which corresponds to 10^8^ cfu/mL.

For the evaluation of MIC the inoculum size for broth dilution is 5 × 10^5^ cfu/mL. The total volume of the culture solution treated by **1**–**2**, SLS, and CitH_4_, as well as the total volume of the positive and negative control was 2 mL. The range of concentrations of **2** is 0.2–250μΜ, for **1** is 50–250μΜ, and SLS or CitH_4_ is 0.5–100μΜ. The growth is assessed after incubation for 20 h.

For the evaluation of MBC the bacteria were initially cultivated in the presence of **1**-**2**, SLS, and CitH_4_, in broth culture for 20 h. The MBC values were determined in duplicate, by subculturing 4 μL of the broth an agar plate [9,11,20,21].

The study of IZ agar plates were inoculated with a standardized inoculum (10^8^cfu/mL) of the tested microorganism. Filter paper disks (9 mm in diameter), which have been previously soaked by **1**–**2**, SLS and CitH_4_, (1 mΜ), were placed on the agar surface. The Petri dishes were incubated for 20 h. The antimicrobial activity of **3** was performed as previously reported [9].

## Figures and Tables

**Table 1:** Minimum Inhibitory Concentrations, Bactericidal Concentrations and Inhibition zones of **1**, **2**, SLS, and CitH_4_ against *St. epidermidis*, *St. aureus*, *PAO1*, and *E. coli*.

Compound	Gram-Positive		Gram-Negative	
	*St. epidermidis*	*St. aureus*	*PAO1*	*E. coli*
	**MIC (μΜ)**			
**1**	119.2 ± 18.1	183.3 ± 31.4	>250	>250
**2**	11.7 ± 2.1	8.5 ± 0.3	228.5 ± 4.6	14.9 ± 0.4
**SLS**	49.7 ± 0.4	42.9 ± 6.2	>250	39.8 ± 0.6
**CitH_4_**	>250	>250	>250	>250
	**MBC (μΜ)**			
**1**	201.7	>250	>250	>250
**2**	13.4	12	>250	>250
**SLS**	100	60	>250	>250
**CitH_4_**	>250	>250	>250	>250
	**MBC/MIC**			
**1**	1.69	ND	ND	ND
**2**	1.15	1.41	ND	ND
**SLS**	2.01	1.40	ND	ND
**CitH_4_**	ND	ND	ND	ND
	**IZ (mm)**			
**1**	ND	ND	ND	ND
**2**	23.0 ± 2.0	30.5 ± 5.4	ND	26.5 ± 1.3
**SLS**	ND	ND	ND	ND
**CitH_4_**	ND	ND	ND	ND
**3**	14.0 ± 2.8	17.0 ± 1.4	ND	ND

ND= No inhibitory zone (IZ) was developed. MIC—minimum inhibitory concentration, MBC—minimum bactericidal concentration.

## Supplementary Materials

The following are available online at https://www.mdpi.com/2079-6382/8/4/213/s1, Figure S1. FT-IR spectra of 1 and CitHNa_3_; Figure S2. UV-Vis spectra of **1** 4.5 ×10^−4^ M in water at 0 and 24 h; Figure S3. CMC determination for the surfactant SLS via conductivity in presence of **1**; Figure S4. FT-IR spectra of **1**, **2** and SLS; Figure S5. TG-DTA curve of **2** (A) and **SLS** (B); Figure S6. Minimum Inhibitory Concentration of **1** (A), **2** (B), SLS (C) and CitH_4_(D) against *St. epidermidis*; Figure S7. Minimum Inhibitory Concentration of **1** (A), **2** (B),SLS (C), CitH_4_ (D) against *St. aureus*; Figure S8. Minimum Inhibitory Concentration of of **1** (A), **2** (B),SLS (C), CitH_4_ (D) against *PAO1*; Figure S9. Minimum Inhibitory Concentration of of **1** (A), **2** (B), SLS (C), CitH_4_ (D) against *E. coli*; Figure S10. Minimum bactericidal concentration of **1** (A), **2** (B), SLS (C), CitH_4_ (D) against *St. epidermidis*.; Figure S11. Results from MBC assay with of **1** (A), **2** (B), SLS (C), CitH_4_ (D) against *St. aureus*; Figure S12. Results from MBC assay with of **1** (A), **2** (B), SLS (C), CitH_4_ (D) against *PAO1*; Figure S13. Results from MBC assay with of **1** (A), **2** (B), SLS (C), CitH_4_ (D) against *E. coli*; Figure S14. Biofilm inhibition of *St. aureus* versus increasing concentrations of **2**. The trend line function that fits better to the points (higher R^2^ parameter) from which the BEC value is determined, is also shown in this figure.
