# Supplementary material for: An Efficient Disinfectant, Composite Material {SLS@[Zn3(CitH)2]} as Ingredient for Development of Sterilized and Non Infectious Contact Lens
Source: Antibiotics (Basel). 2019 Nov 7;8(4):213. doi: 10.3390/antibiotics8040213 (PMC6963967; doi:10.3390/antibiotics8040213)
Supplement: Supplementary file 1 [file antibiotics-08-00213-s001.pdf]

antibiotics

Supplementary Information

An Efficient Disinfectant, Composite Material {SLS@[$Zn_3(CitH)_2$]} as Ingredient for Development of Sterilized and non Infectious Contact Lens

V.A. Karetsi[1], C.N. Banti[1,*], N. Kourkoumelis[2], C. Papachristodoulou[3], C.D. Stalikas[4], C.P. Raptopoulou[5], V. Psycharis[5], P. Zoumpoulakis[6], T. Mavromoustakos[7], I. Sainis[8] and S.K. Hadjikakou[1,*]

[1] Inorganic Chemistry laboratory, Department of Chemistry, University of Ioannina, 45110 Ioannina, Greece; basilikikaretsi92@gmail.com
[2] Medical Physics Laboratory, Medical School, University of Ioannina, 45110 Ioannina, Greece; nkourkou@uoi.gr
[3] Department of Physics, University of Ioannina, 45110 Ioannina, Greece; xpapaxri@uoi.gr
[4] Laboratory of Analytical Chemistry, Department of Chemistry, University of Ioannina, 45110 Ioannina, Greece; cstalika@uoi.gr
[5] Institute of Nanoscience and Nanotechnology, NCSR "Demokritos", 15341 Athens, Greece; c.raptopoulou@inn.demokritos.gr (C.P.R.); v.psycharis@inn.demokritos.gr (V.P.)
[6] Institute of Biology, Medicinal Chemistry and Biotechnology, National Hellenic Research Foundation, 11635 Athens, Greece; pzoump@eie.gr
[7] Organic Chemistry Laboratory, Department of Chemistry, National and Kapodistrian University of Athens, 15571 Athens, Greece; tmavrom@chem.uoa.gr
[8] Cancer Biobank Center, University of Ioannina, 45110 Ioannina, Greece; isainis@cc.uoi.gr
* Correspondence: cbanti@uoi.gr (C.N.B.), shadjika@uoi.gr (S.K.H.); Tel.: +30-26510-08374 (S.K.H.)

antibiotics

MDPI

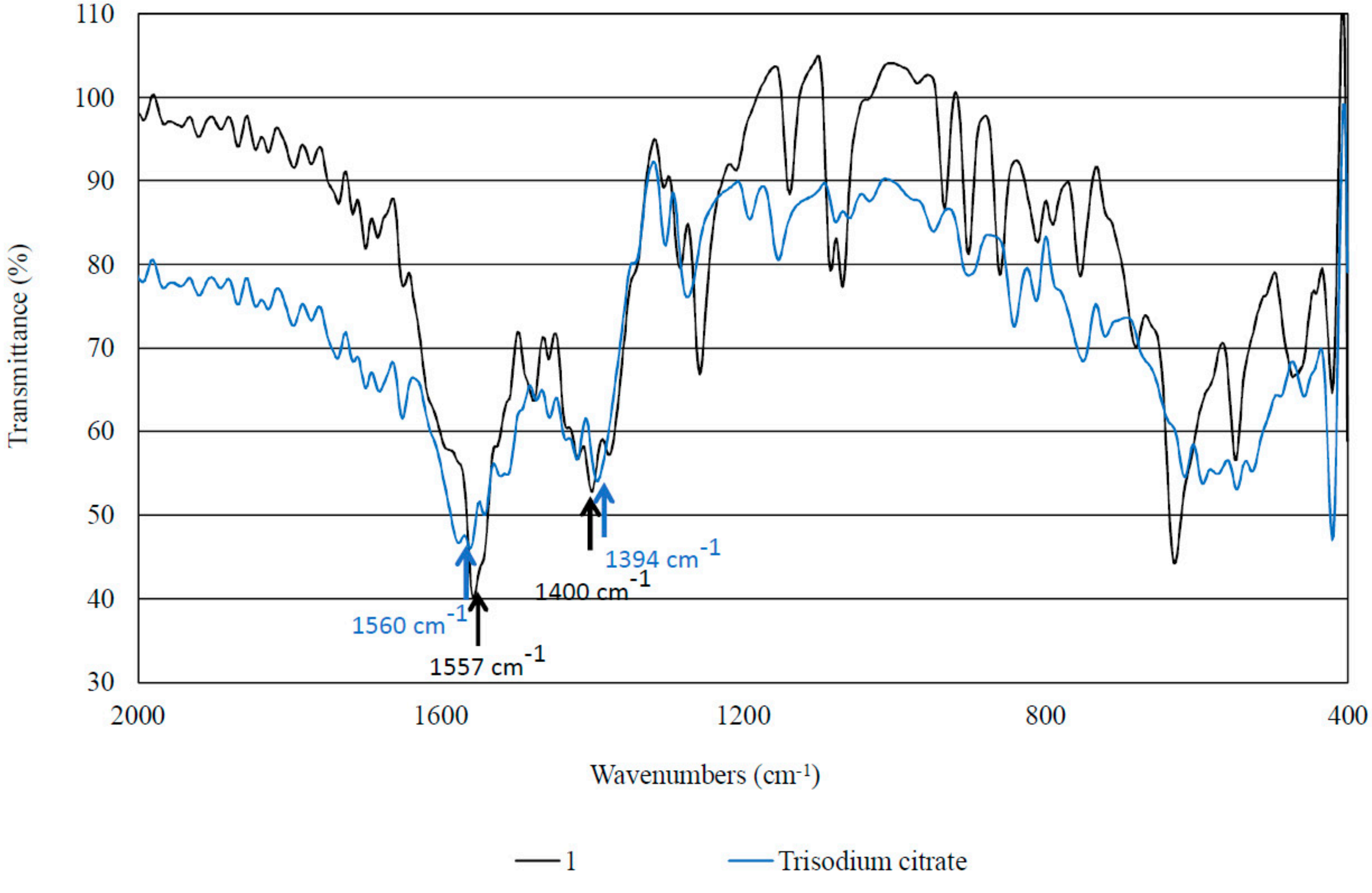

Figure S1. FT-IR spectra of **1** and CitHNa3.

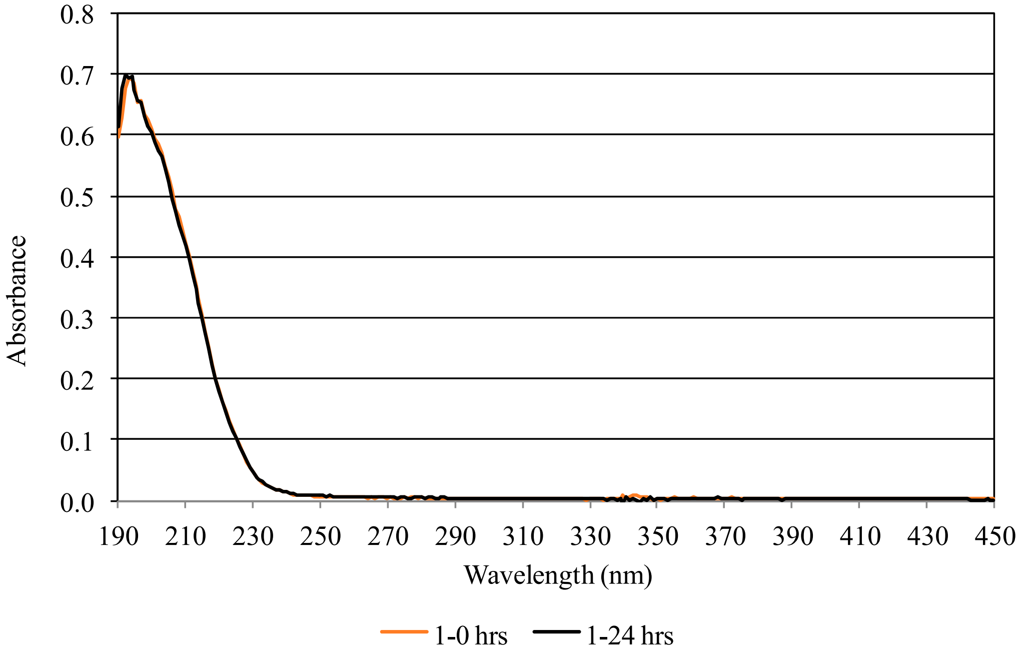

Figure S2. UV-Vis spectra of **1** 4.5 ×10^{-4} M in water at 0 and 24 h

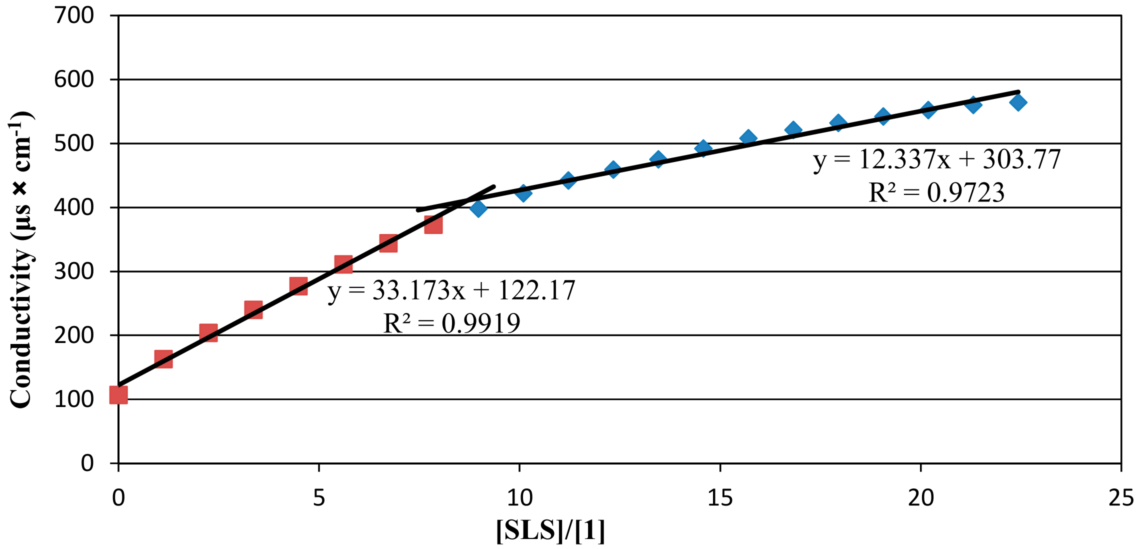

Figure S3. CMC determination for the surfactant SLS via conductivity in presence of **1**

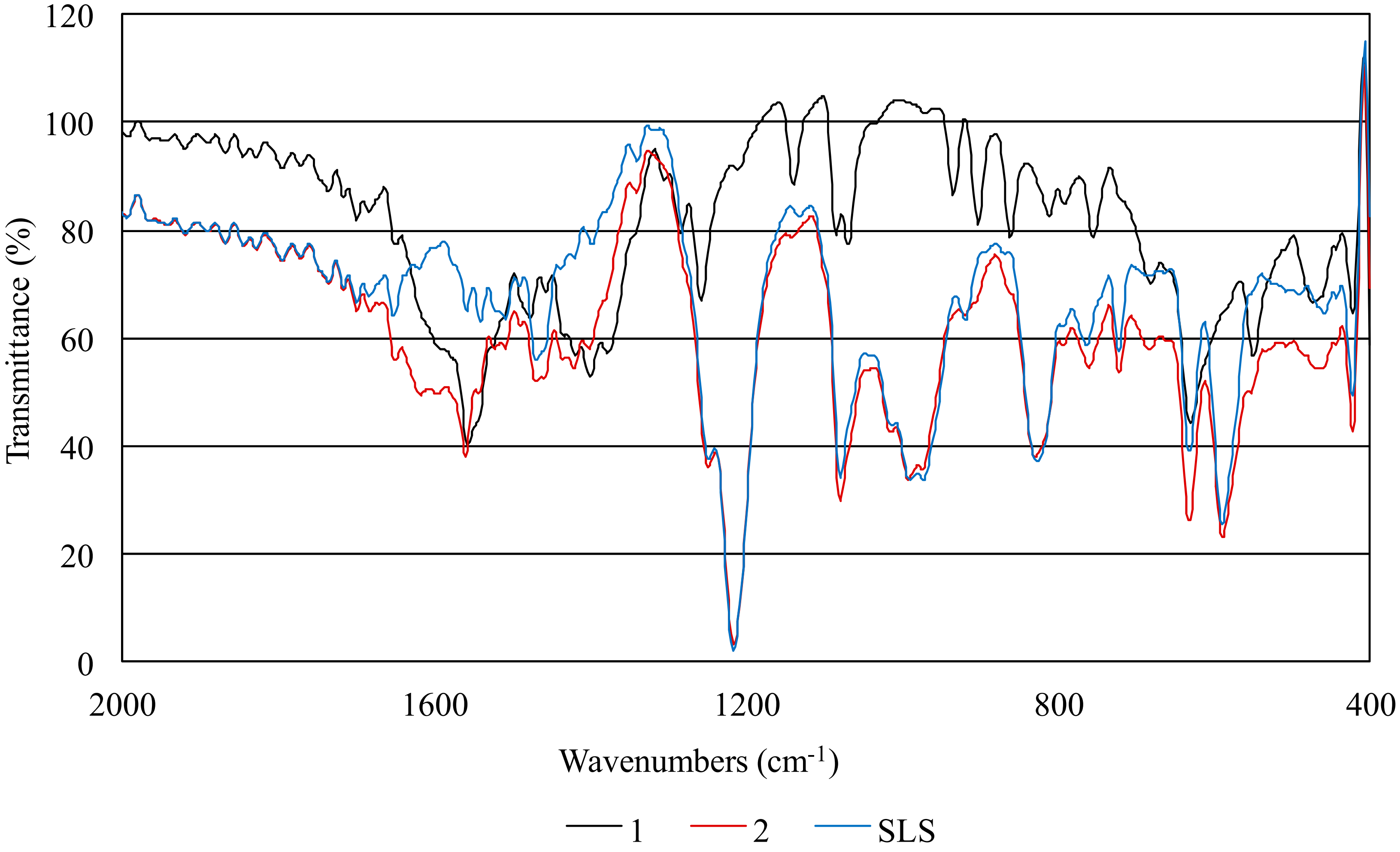

Figure S4. FT-IR spectra of **1**, **2** and **SLS**

antibiotics

MDPI

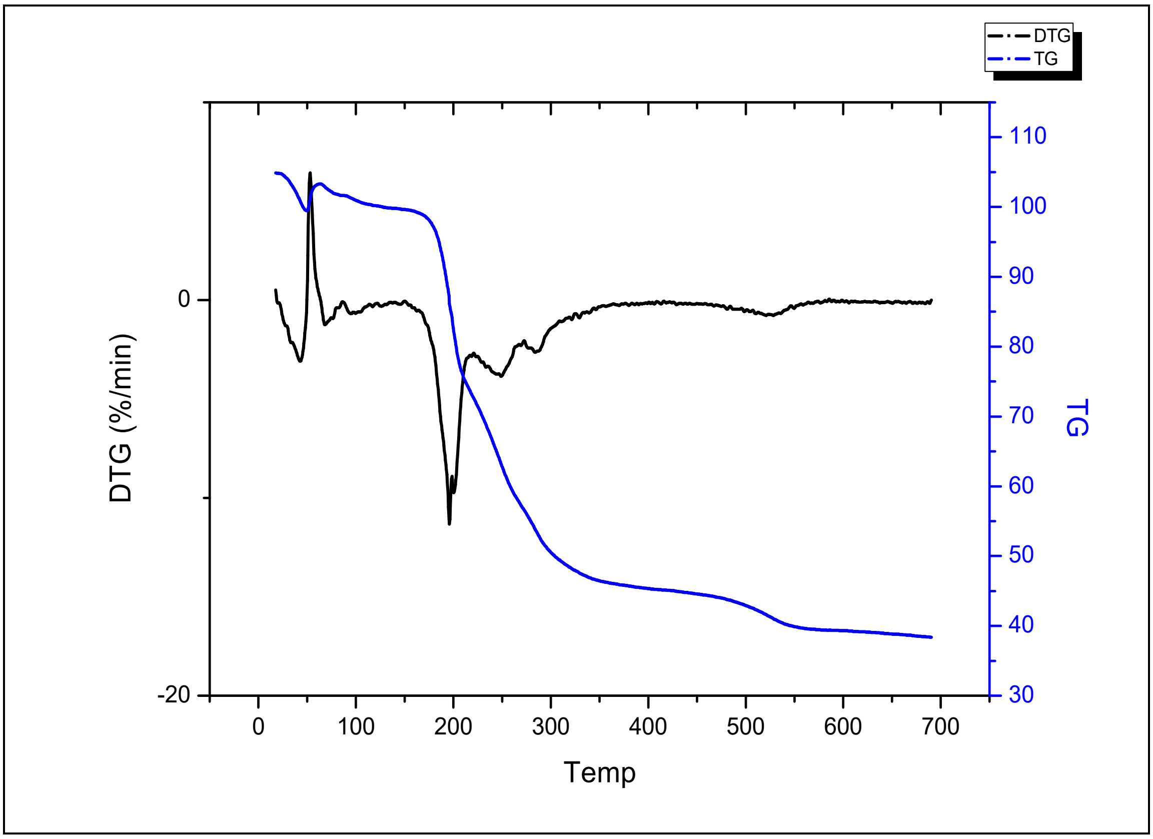

(A)

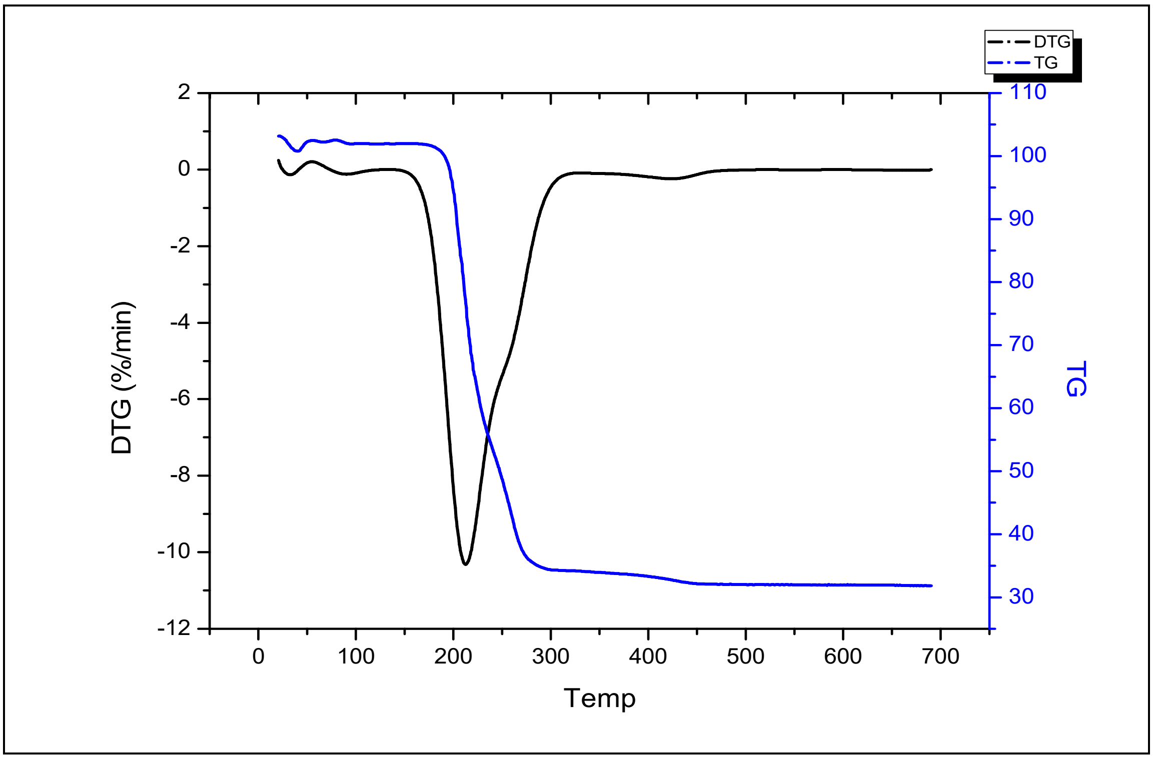

(B)

Figure S5 . TG-DTA curve of **2** (**A**) and **SLS** (**B**)

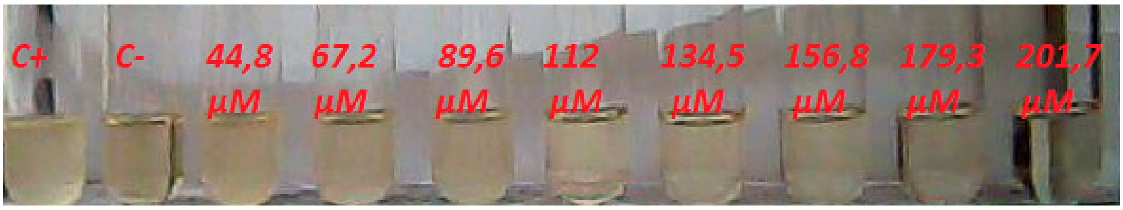

(A)

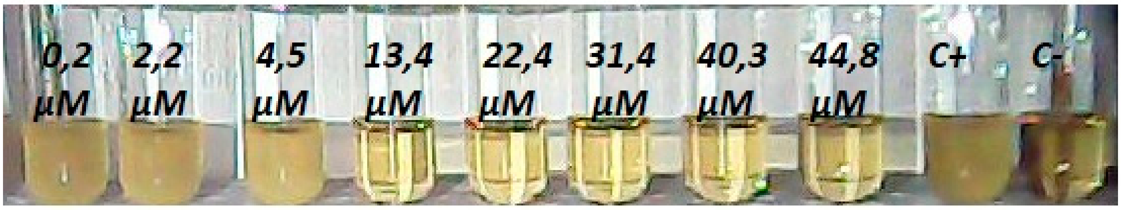

(B)

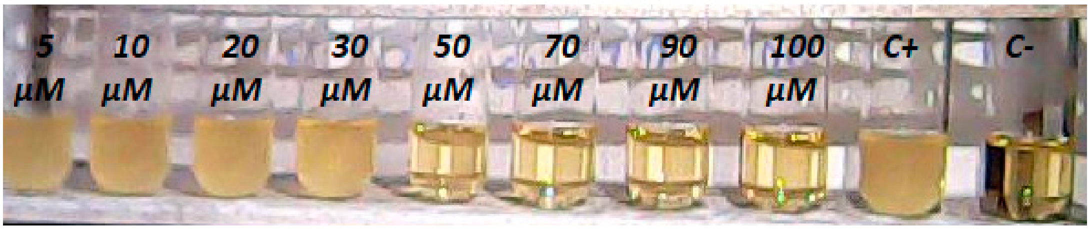

(C)

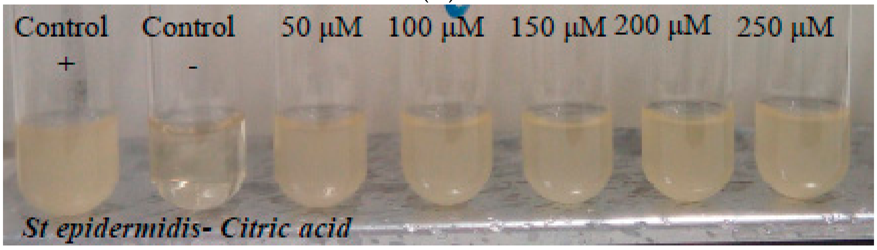

(D)

Figure S6. Minimum Inhibitory Concentration of **1** (**A**), **2** (**B**), **SLS** (**C**) and $CitH_4$ (**D**) against *St. epidermidis*

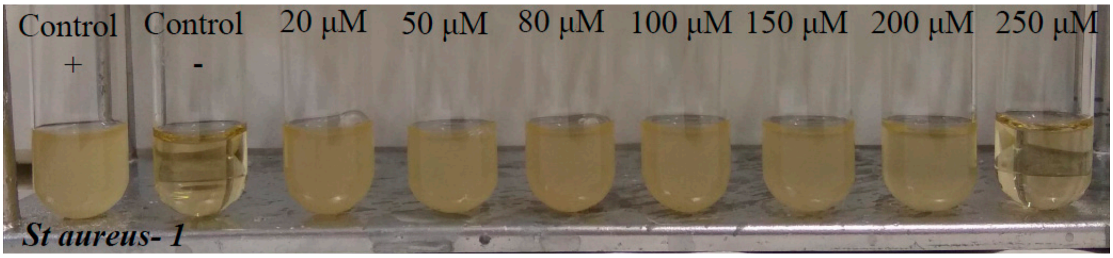

(A)

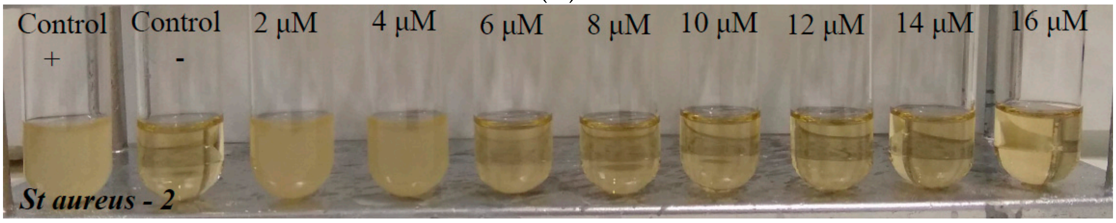

(B)

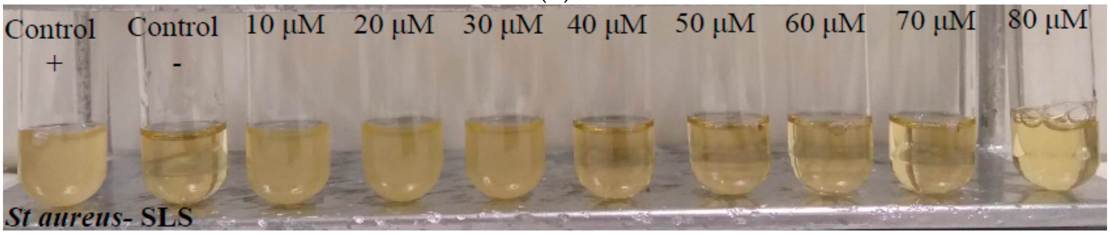

(C)

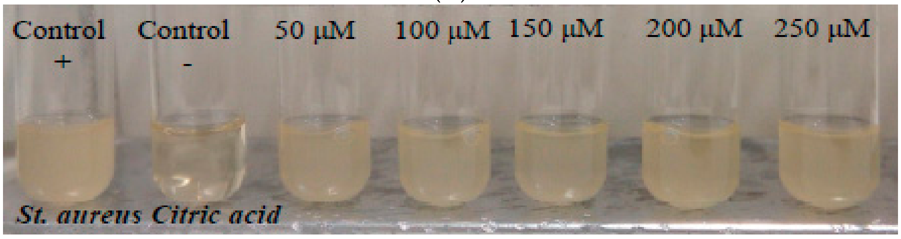

(D)

Figure S7. Minimum Inhibitory Concentration of of **1** (**A**), **2** (**B**), **SLS** (**C**) and $CitH_4$ (**D**) against *St. aureus*

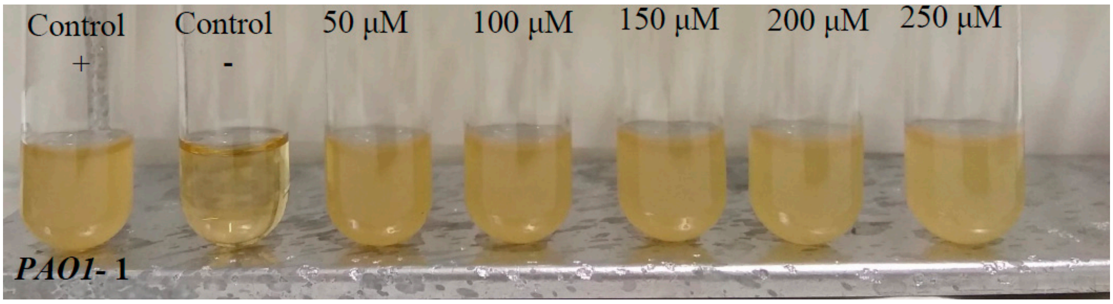

(A)

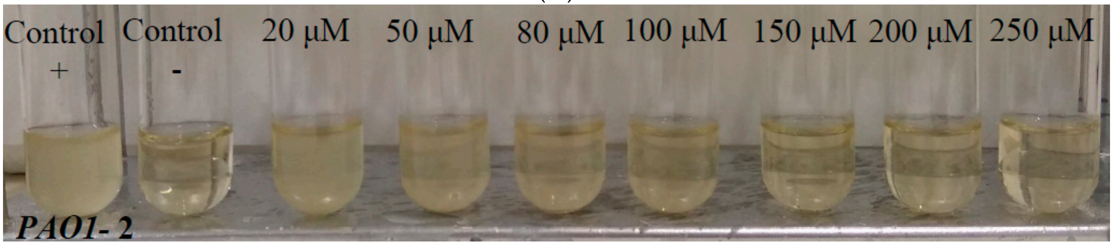

(B)

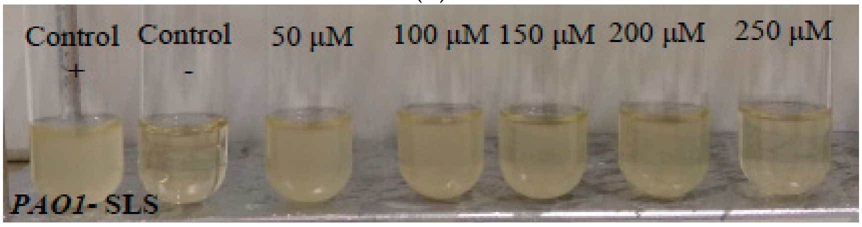

(C)

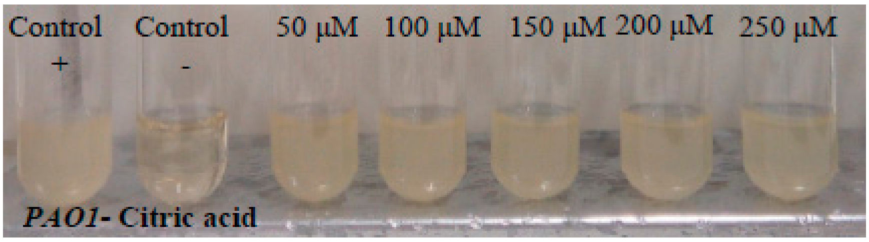

(D)

Figure S8. Minimum Inhibitory Concentration of of **1** (**A**), **2** (**B**), **SLS** (**C**) and $CitH_4$ (**D**) against *PAO1*

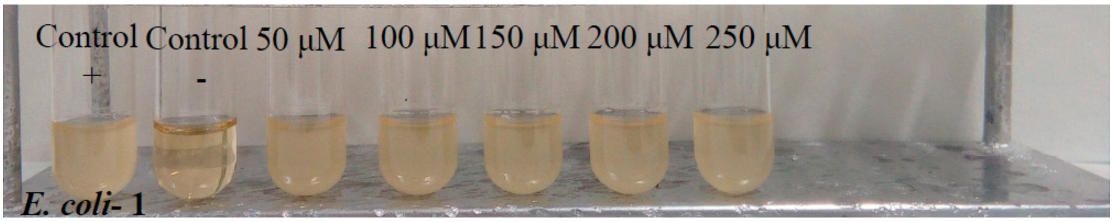

(A)

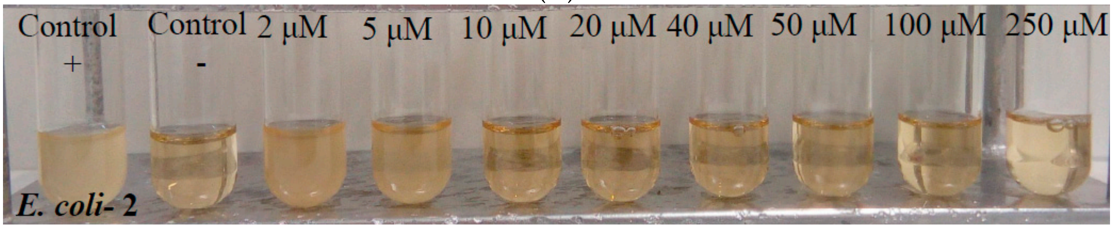

(B)

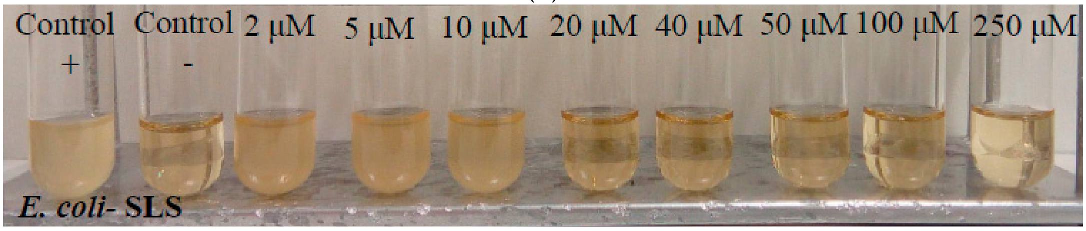

(C)

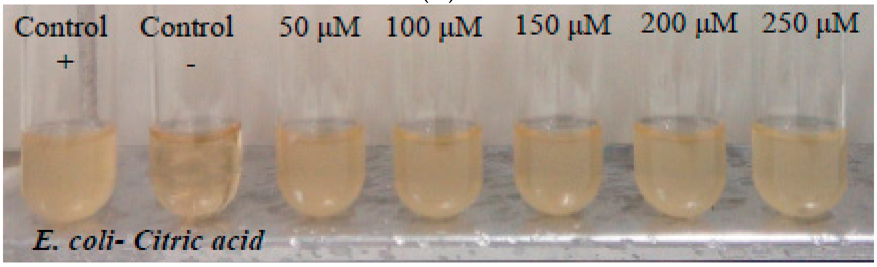

(D)

Figure S9. Minimum Inhibitory Concentration of of **1** (**A**), **2** (**B**), **SLS** (**C**) and $CitH_4$ (**D**) against *E. coli*

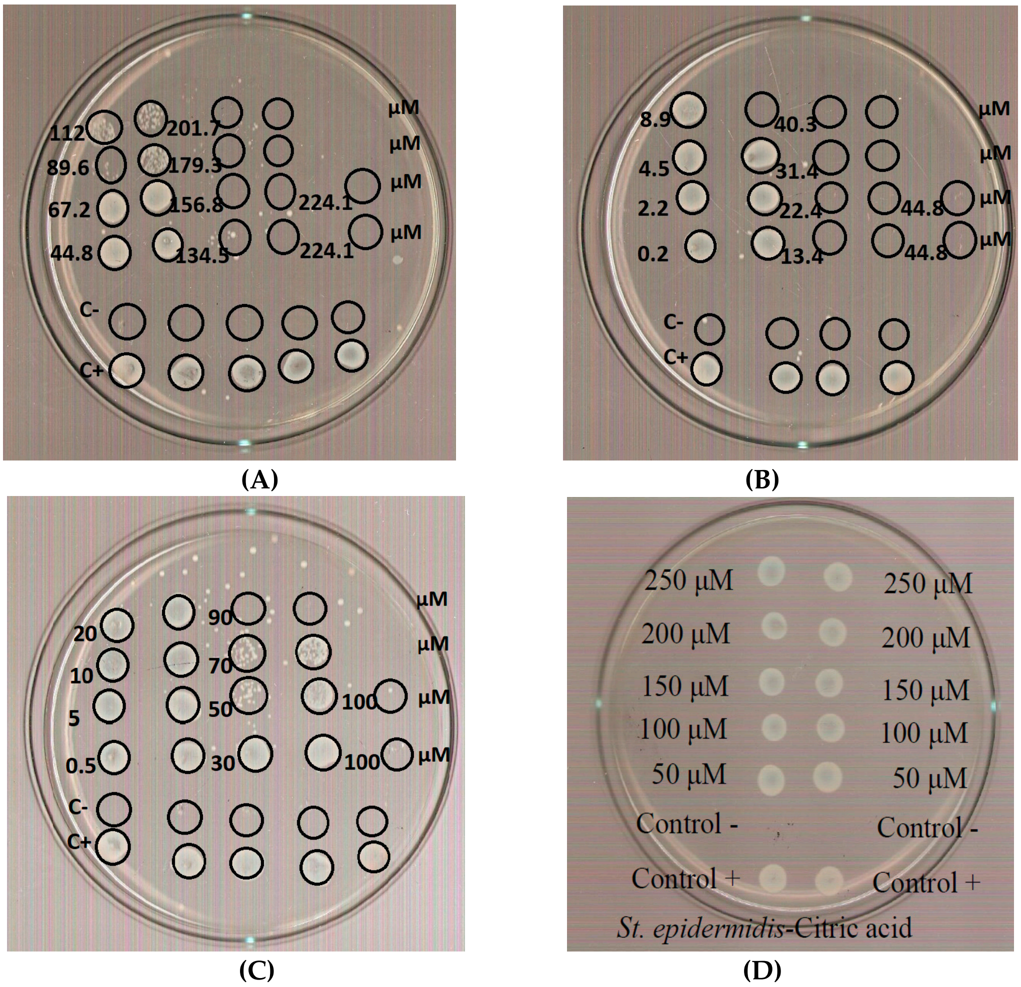

Figure S10. Minimum bactericidal concentration of **1** (**A**), **2** (**B**), **SLS** (**C**) and $CitH_4$ (**D**) against *St. epidermidis*.

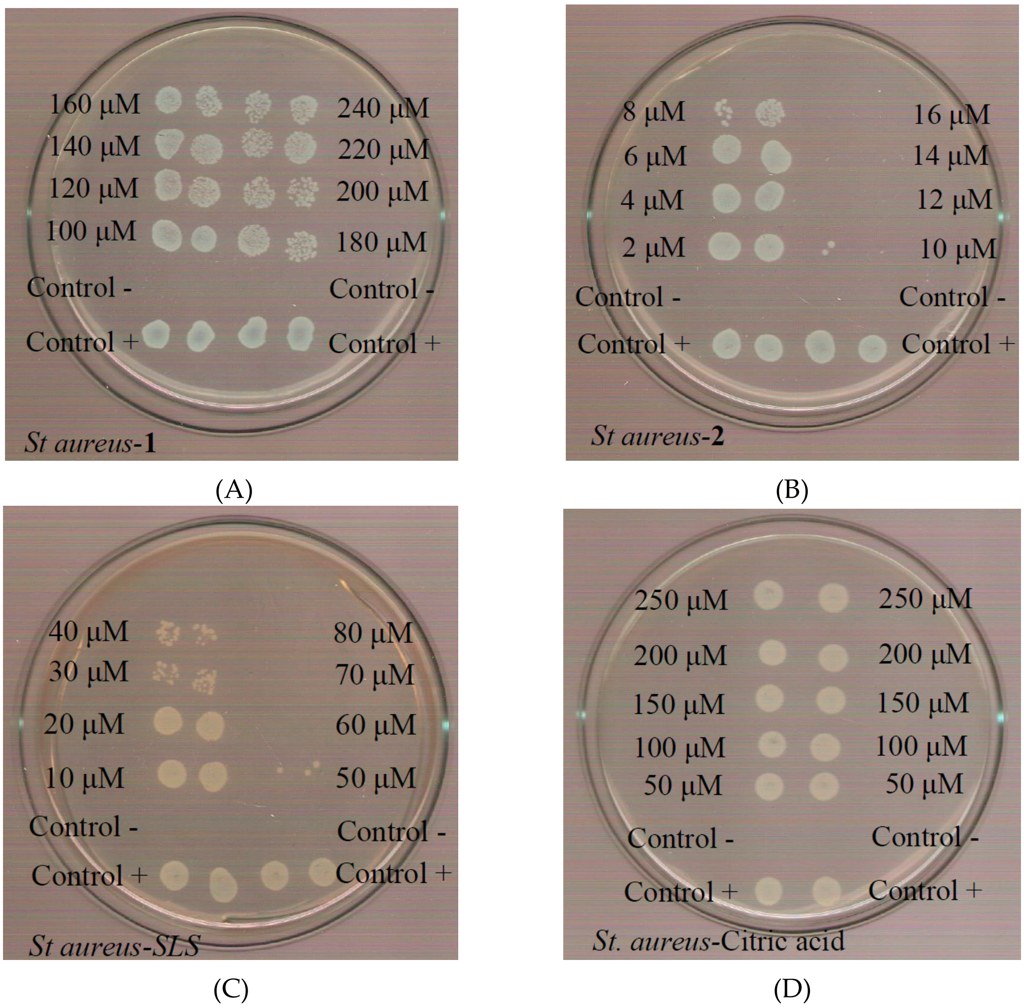

Figure S11. Results from MBC assay with of **1** (**A**), **2** (**B**), **SLS** (**C**) and $CitH_4$ (**D**) against and *St. aureus*

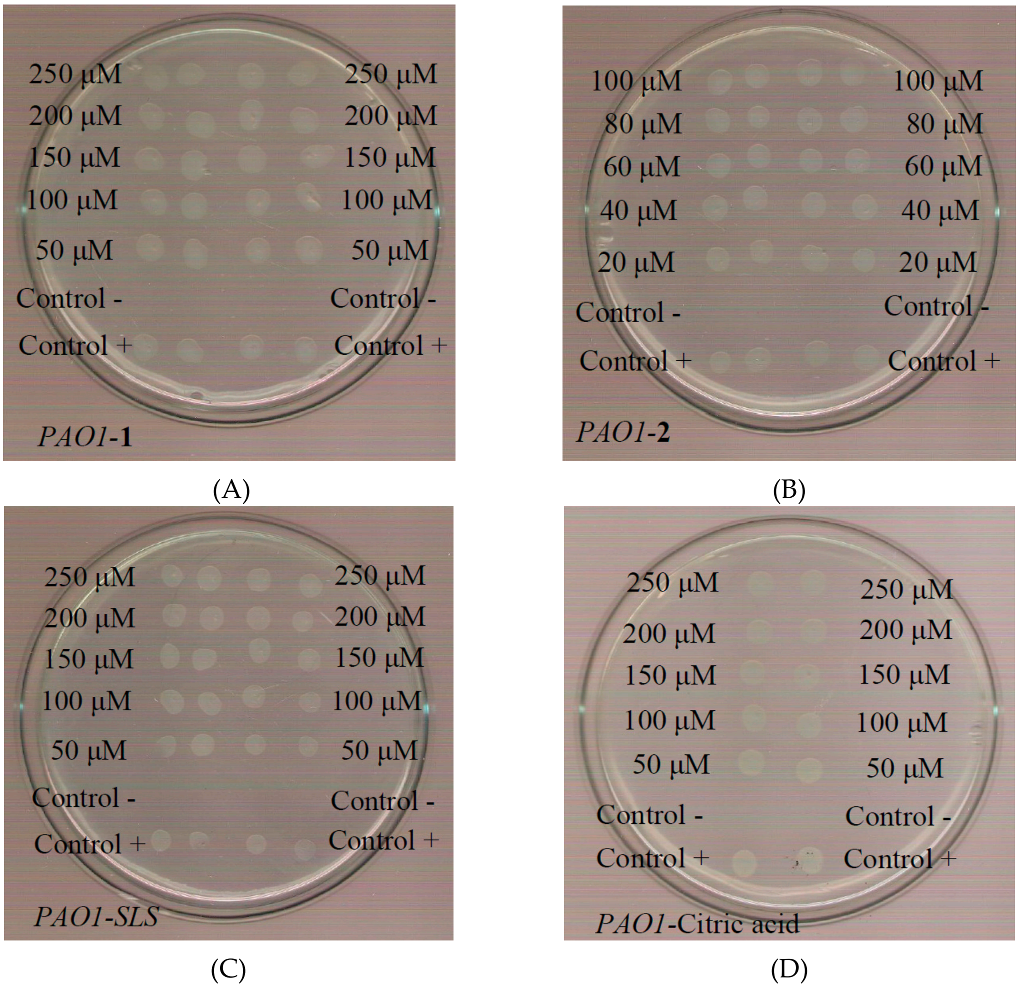

Figure S12. Results from MBC assay with of **1** (**A**), **2** (**B**), **SLS** (**C**) and $CitH_4$ (**D**) against and *PAO1*

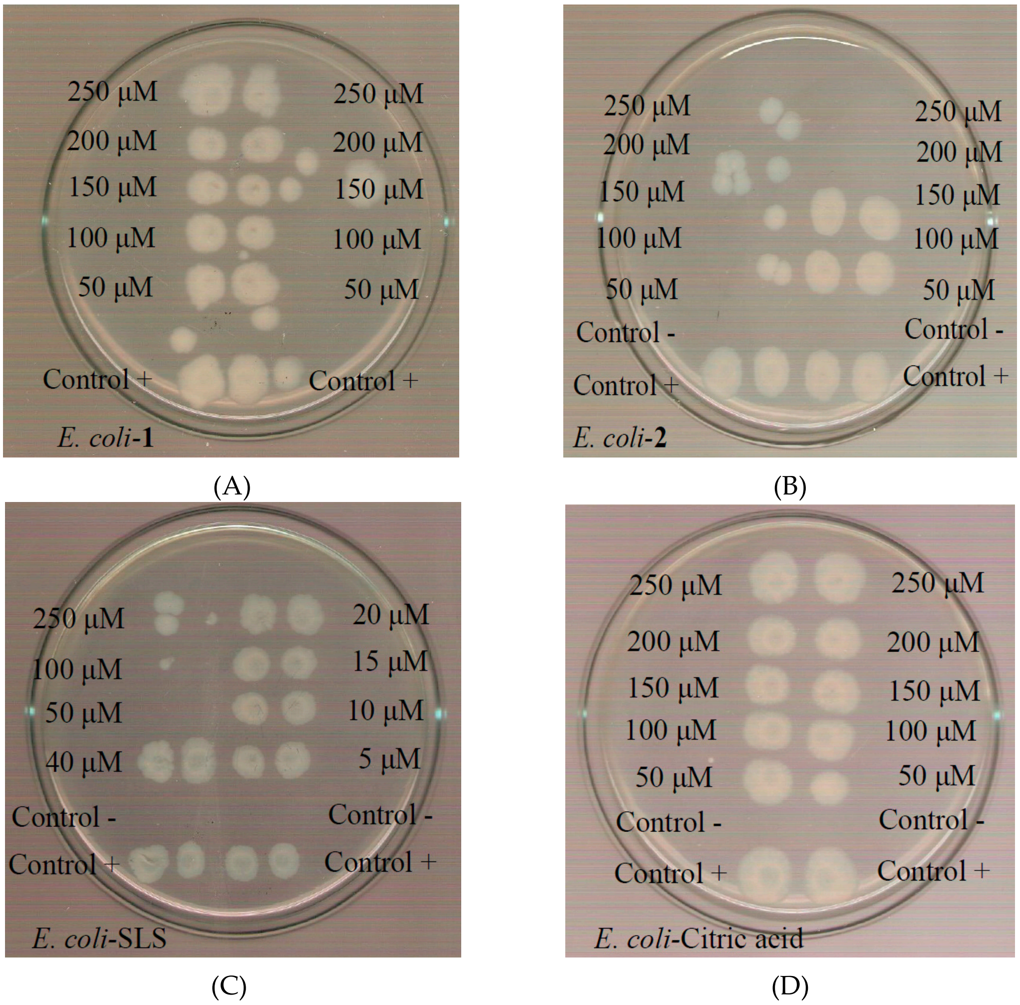

Figure S13. Results from MBC assay with of **1** (**A**), **2** (**B**), **SLS** (**C**) and $CitH_4$ (**D**) against and *E. coli*

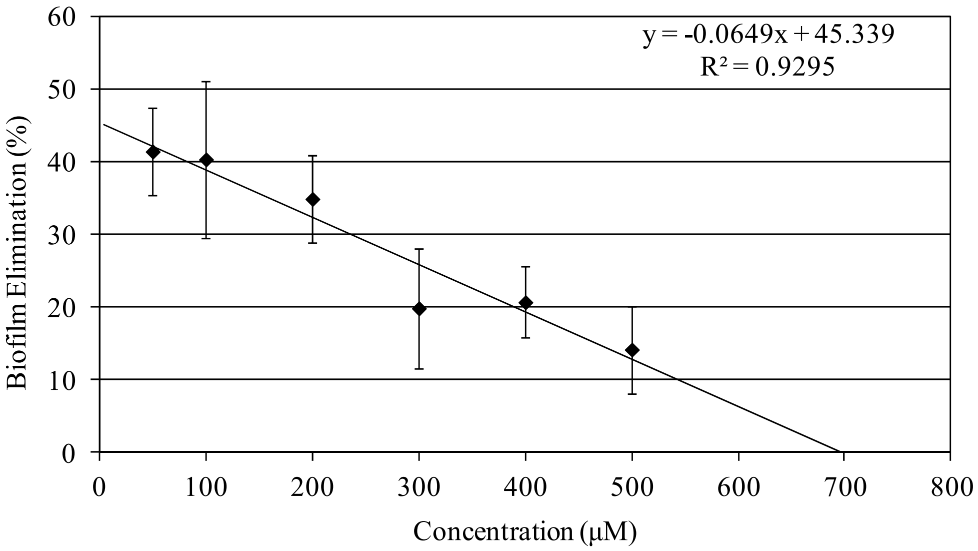

Figure S14. Biofilm inhibition of *St. aureus* versus increasing concentrations of **2**. The trend line function that fits better to the points (higher R^2 parameter) from which the BEC value is determined, is also shown in this figure.
